# Cross-species transcriptomes reveal species-specific and shared molecular adaptations for plants development on iron-rich rocky outcrops soils

**DOI:** 10.1186/s12864-022-08449-0

**Published:** 2022-04-19

**Authors:** Mariana Costa Dias, Cecílio Caldeira, Markus Gastauer, Silvio Ramos, Guilherme Oliveira

**Affiliations:** 1 Instituto Tecnológico Vale, Rua Boaventura da Silva 955, Belém, Pará CEP 66055-090 Brazil; 2 Universidade Federal de Minas Gerais, Avenida Antônio Carlos 6627, Belo Horizonte, Minas Gerais CEP 31270-901 Brazil

**Keywords:** *Caesalpinioideae*, *Canga*, Comparative transcriptomics, De novo transcriptome, *Fabaceae*, Gene expression plasticity, Ironstone outcrops, *Leguminosae*, RNA-seq

## Abstract

**Background:**

*Canga* is the Brazilian term for the savanna-like vegetation harboring several endemic species on iron-rich rocky outcrops, usually considered for mining activities. *Parkia platycephala* Benth. and *Stryphnodendron pulcherrimum* (Willd.) Hochr. naturally occur in the *cangas* of Serra dos Carajás (eastern Amazonia, Brazil) and the surrounding forest, indicating high phenotypic plasticity. The morphological and physiological mechanisms of the plants’ establishment in the *canga* environment are well studied, but the molecular adaptative responses are still unknown. To understand these adaptative responses, we aimed to identify molecular mechanisms that allow the establishment of these plants in the *canga* environment.

**Results:**

Plants were grown in *canga* and forest substrates collected in the Carajás Mineral Province. RNA was extracted from pooled leaf tissue, and RNA-seq paired-end reads were assembled into representative transcriptomes for *P. platycephala* and *S. pulcherrimum* containing 31,728 and 31,311 primary transcripts, respectively. We identified both species-specific and core molecular responses in plants grown in the *canga* substrate using differential expression analyses. In the species-specific analysis, we identified 1,112 and 838 differentially expressed genes for *P. platycephala* and *S. pulcherrimum,* respectively. Enrichment analyses showed that unique biological processes and metabolic pathways were affected for each species. Comparative differential expression analysis was based on shared single-copy orthologs. The overall pattern of ortholog expression was species-specific. Even so, we identified almost 300 altered genes between plants in *canga* and forest substrates with conserved responses in the two species. The genes were functionally associated with the response to light stimulus and the circadian rhythm pathway.

**Conclusions:**

Plants possess species-specific adaptative responses to cope with the substrates. Our results also suggest that plants adapted to both *canga* and forest environments can adjust the circadian rhythm in a substrate-dependent manner. The circadian clock gene modulation might be a central mechanism regulating the plants’ development in the *canga* substrate in the studied legume species. The mechanism may be shared as a common mechanism to abiotic stress compensation in other native species.

**Supplementary Information:**

The online version contains supplementary material available at 10.1186/s12864-022-08449-0.

## Background

Outcrops of banded iron formations found in the Serra dos Carajás (Pará, Northern Brazil) typically occur in iron-rich areas worldwide [1]. They are covered by diverse savanna-like vegetation known in Brazil as *canga*, harboring many endemic species. These challenging environments are characterized by high temperatures and UV radiation, together with a strong seasonal water regime, shallow and acidic soils, with low nutrients availability (especially phosphorus, magnesium, and calcium), and high total metal concentrations (such as iron and manganese) [2–4].

The *cangas* of Serra dos Carajás are inserted in the Amazon Rainforest, rising abruptly from the surrounding lowland vegetation matrix. Plant species of ironstone outcrops grow in high-stress habitats that restrict the species composition. This result in structurally and floristically distinct vegetation from the surrounding forest, with many species being specific to the *canga* environment [5]. Nevertheless, some species occur in both ecosystems [5], tolerating a wide range of conditions and displaying high phenotypic plasticity [6, 7].

The main characteristics of plants adapted to the *canga* environment involve features such as thick, waxy, coriaceous, and hairy leaves; protected stomata and stomata activity control; idioblasts containing phenolic compounds and crystals and the presence of water-storing parenchymatous tissues [2, 3, 8]. The *canga* plant community also presents metal tolerance by excluding or accumulating (or hyper-accumulating) metals in their shoots [2, 3, 9]. Some studies also demonstrated the importance of soil microorganisms in the maintenance and growth of plant species in metal-rich *canga* environments [10–12]. Although several species in the Carajás' *cangas* shows metal accumulation [9], there is no study evaluating the plants' adaptive genetic responses that allows them to thrive in such a stressful environment.

*Fabaceae*, also known as *Leguminosae*, comprises several socioeconomic crops, being the second most cultivated plant family [13]. Moreover, it is also one of the most prominent plant families found in the Carajás *cangas*, containing almost 80 species [14]. The family includes approximately 19,500 species under six recently described subfamilies: *Cercidoideae, Detarioideae, Dialioideae, Duparquetioideae, Papilionoideae*, and *Caesalpinioideae*, which contains the former subfamily *Mimosoidea*e [15]. The most prominent ecological trait of the family *Fabaceae* is the possibility of some legume species to fix efficiently atmospheric nitrogen in symbiosis with soil bacteria [16]. Furthermore, plant-associated bacteria present a potential for rhizoremediation [10, 11]. Thus, the association with bacteria is an advantageous adaptation to occupy extreme environments, such as the *cangas*.

*Parkia platycephala* Benth. and *Stryphnodendron pulcherrimum* (Willd.) Hochr. are two species of tropical *Caesalpinioideae* with distribution ranges comprising different bioclimatic regions, biomes, and habitats, with natural occurrences in *canga* environments (shrublands and woodlands) and surrounding forests. *Parkia platycephala* is endemic to Brazil, occurring in the domains of Amazon, Caatinga, and Cerrado [17], while *S. pulcherrimum* is more widely distributed over the South American continent, covering large phytogeographic domains of Amazon, Caatinga, and Atlantic Forest [17]. Both species are suitable for mine land rehabilitation programs [18–21], exhibiting fast growth rates and the capacity to tolerate drought [19, 22].

Silva and collaborators (2018) [19] evaluated the phenotypic variation in the initial growth of five *Fabaceae* species influenced by different substrates obtained from the Carajás region. *Parkia platycephala* and *S. pulcherrimum* were included in that study because of their wide distribution and occurrence in forest and *canga* ecosystems. The authors found that plants of *P. platycephala* and *S. pulcherrimum* grown in unfertilized *canga* and forest substrates showed no difference in the initial growth rate. Between the two species, only *S. pulcherrimum* exhibited symbiotic interactions with nitrogen-fixing bacteria, with a higher percentage when grown in the forest topsoil. Both *P. platycephala* and *S. pulcherrimum* showed higher foliar concentrations of manganese (Mn) and iron (Fe) and higher relative investment in root development when grown in the *canga* substrate compared to the forest substrate [19].

Environment-induced phenotypic plasticity plays an important role in organisms’ development, function, and adaptation. Gene expression plasticity is the main biological process that induces phenotypic variation [23]. The gene expression responds to environmental cues and differs depending on the developmental stage, cell types, tissues, and organisms [24]. Therefore, studying gene expression through transcriptome sequencing is a powerful approach to quantifying differentially expressed genes and measuring how environmental stress affects gene activity. Several plant transcriptome studies identified genes that respond to environmental stresses, such as salt, heat, cold, drought, light, ozone, excessive boron, and pathogen infection [25–27]. Hence, identifying natural genetic resources and characterizing adaptive genetic variation in *canga* plants may increase our knowledge of the genes linked to several abiotic stress such as heavy metals, nutrient deficiency, drought, and heat. This knowledge can be applied to native species in land rehabilitation [21] and can also provide indications of adaptative responses of species with importance to agriculture, thus improving food security of those crops subjected to climate changes [28].

Therefore, to understand how plants can thrive in *canga* environments, we studied the gene expression plasticity when grown in the *canga* soil compared to in the forest using two *Leguminosae* species: *P. platycephala* and *S. pulcherrimum*. The results indicated a species-specific adaptative responses and a core gene set responsive to the *canga* condition that may be shared by other plant species.

## Results

The leguminous species were cultivated in four different substrates collected at the Serra dos Carajás (*canga*, forest, and two mine waste sites) [19]. Plants grown in two trays of each substrate were harvested. Leaf samples from three individuals in the same tray were pooled together for RNA extraction. In the present study, we aimed to assemble high-quality and complete transcriptomes to use as a reference for *P. platycephala* and *S. pulcherrimum*. Furthermore, we sought to reveal the gene expression plasticity of the plants grown in substrates where they naturally occur and to establish if the species exhibit conserved molecular responses to *canga*. We used the well-established differential expression (DE) analyses to reveal the species-specific responses and orthologs DE analysis to test for conserved responses.

### Transcriptome sequencing and de novo assembly

We used high-throughput RNA sequencing (RNA-seq) of leaf samples from plants grown in all four substrates for the reference transcriptomes assembly. The sequencing output produced between 46 to 91 million paired-end reads per sample (See Supplementary Table S1, Additional File 1).

In total, 15 assemblies were generated for *P. platycephala* and 17 for *S. pulcherrimum* (See Supplementary Table S2, Additional File 1), which were then merged into one over-assembly for each species. For both species, Trinity v.2.8.3 produced assemblies with the highest average transcript length, highest N50 value, and highest number of assembled bases (See Supplementary Table S2, Additional File 1). SOAPdenovo-Trans v.1.03 had the longest transcripts (37,652 bp with k-mer size 61 for *P. platycephala* and 49,517 bp with k-mer size 27 for *S. pulcherrimum*), but also the highest number of transcripts for both species. The number of contigs ranged from 121,698 and 94,733 with Velvet v.1.2.10/Oases v.0.2.09 using a k-mer size of 61 to 255,644 and 233,410 with SOAPdenovo-Trans and a k-mer of 31 for *P. platycephala* and *S. pulcherrimum*, respectively (See Supplementary Table S2, Additional File 1).

The over-assembly approach enabled the recovery of many potential transcripts and their variants but resulted in very large assemblies containing many redundant sequences (Table 1). *P. platycephala* and *S. pulcherrimum* over-assemblies included 2,794,335 and 2,898,193 de novo assembled transcripts, respectively (See Supplementary Table S2, Additional File 1). The large assemblies were reduced with the EvidentialGene pipeline v.4. They resulted in 405,001, and 393,111 de novo assembled transcripts for *P. platycephala* and *S. pulcherrimum*, with 84,422 and 85,521 classified as the main set of transcripts (primary transcripts), respectively.

**Table 1:** BUSCO results from the de novo transcriptomes of *P. platycephala* and *S. pulcherrimum*

BUSCOs	*Parkia platycephala*			*Stryphnodendron pulcherrimum*		
Embryophyta / Eudicotyledons	Over-assembly	Evigene reduced assembly	Main filtered set	Over-assembly	Evigene reduced assembly	Main filtered set
Complete	1358 (94.3%)	1357 (94.3%)	1314 (91.2%)	1355 (94.1%)	1354 (94.0%)	1312 (91.1%)
	2041 (96.2%)	2046 (96.5%)	2003 (94.5%)	2027 (95.6%)	2037 (96.0%)	1981 (93.4%)
Complete and single-copy	50 (3.5%)	214 (14.9%)	1201 (83.4%)	34 (2.4%)	205 (14.2%)	1236 (85.8%)
	70 (3.3%)	311 (14.7%)	1840 (86.8%)	47 (2.2%)	312 (14.7%)	1862 (87.8%)
Complete and duplicated	1308 (90.8%)	1143 (79.4%)	113 (7.8%)	1321 (91.7%)	1149 (79.8%)	76 (5.3%)
	1971 (92.9%)	1735 (81.8%)	163 (7.7%)	1980 (93.4%)	1725 (81.3%)	119 (5.6%)
Fragmented	28 (1.9%)	28 (1.9%)	44 (3.1%)	22 (1.5%)	21 (1.5%)	24 (1.7%)
	41 (1.9%)	34 (1.6%)	52 (2.5%)	39 (1.8%)	31 (1.5%)	52 (2.5%)
Missing	54 (3.8%)	55 (3.8%)	82 (5.7%)	63 (4.4%)	65 (4.5%)	104 (7.2%)
	39 (1.9%)	41 (1.9%)	66 (3.0%)	55 (2.6%)	53 (2.5%)	88 (4.1%)

The de novo transcriptomes of *P. platycephala* and *S. pulcherrimum* were compared to the embryophyta (upper lines) and the eudicotyledons (bottom lines) databases. The percentage of inferred orthologs is in parentheses next to the number of orthologs

The Evigene draft transcript sets for *P. platycephala* and *S. pulcherrimum* are 7 × above other *Caesalpinioideae* species set counts [29]. Almost all the excess were unclassified short proteins. Of the main set, 28,561 (*P. platycephala*) and 27,632 (*S. pulcherrimum*) predicted proteins contained 120 amino acids (120aa) or more. As short putative proteins may be spurious loci, from the main set of transcripts, 55,861 and 57,889 short predicted proteins were blasted (e-value 1 × 10^–6^) against the UniProtKB/Swiss-Prot *Viridiplantae* database to attempt to establish homology evidence for them. Only 3,167 and 3,679 short proteins for *P. platycephala* and *S. pulcherrimum,* respectively, had hits with the *Viridiplantae* database and were kept for downstream analysis. The remaining short proteins and their alternative forms were discarded. From now on, primary transcripts with predicted proteins of 120aa or longer and the short ones with similarity to the *Viridiplantae* database will be referred to as the main filtered transcripts.

The differential expression (DE) analyses were performed with the samples from the *canga* and forest substrates to reveal the gene expression plasticity of the plants grown in substrates where they naturally occur. The main filtered transcripts added to their alternative forms were used for the species-specific DE analysis. The main filtered predicted proteins were used for orthology prediction. The single-copy orthologs were submitted to DE analysis between the substrates to test whether the species exhibit conserved molecular responses to the *canga* substrate.

### Quality check and gene orthology prediction

We evaluated the rate of remapping and found that samples were aligned with their respective transcriptomes with mapping rates of 98.98% for *P. platycephala* and 99.19% for *S. pulcherrimum*.

To assess the completeness of the assemblies, we used the Benchmarking Universal Single-Copy Ortholog (BUSCO) assessment tool. The plant transcriptomes were compared against the embryophyta and the eudicotyledons single-copy orthologs database. We achieved over 94% of the complete single-copy genes of embryophyta and eudicotyledons orthologs with BUSCO evaluation for both transcriptomes (Table 1). Many of these genes were duplicated in the assemblies, even after the EvidentialGene reduction, due to the presence of multiple isoforms. The BUSCO analysis with the main filtered transcripts (which excluded alternative isoforms) of the two species showed a marked decrease of the duplicated genes (from approximately 80% to 7% and 5%, for *P. platycephala* and *S. pulcherrimum,* respectively), maintaining at over 90% the recovery of complete orthologs of both embryophyta and eudicotyledons (Table 1). Additionally, only between 1.9% and 7.2% of near-universal genes were classified as missing in the plants’ transcriptomes in all BUSCO analyses, indicating the transcriptomes' high quality and good coverage.

**Fig. 1:**
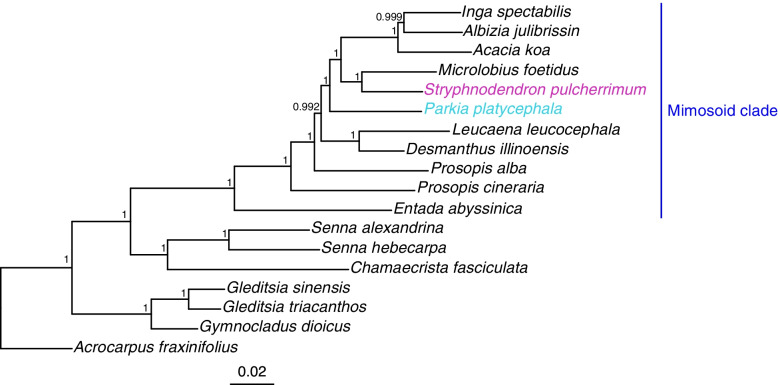
Approximate Maximum Likelihood phylogenetic tree based in single-copy orthologs of *Caesalpinioideae* species, generated with OrthoFinder. The branch points' numbers represent support values based on 1,000 local bootstrapping resamples generated with FastTree

To further evaluate the transcriptome assemblies, we examined the molecular phylogeny of the *Caesalpinioideae* subfamily. OrthoFinder v.2.3.12 was used to detect putative orthologs and orthology grouping using the two transcriptomes' main filtered proteins and the predicted proteins of 16 genome or transcriptome sequences of other *Caesalpinioideae* species downloaded from multiple sources (See Supplementary Table S3, Additional File 1). As expected, the ortholog phylogram shows the two species' placement within the Mimosoid clade and the relative proximity of each other (Fig. 1). Both species were part of a subclade composed of the genera *Inga*, *Albizia*, *Acacia,* and *Microlobius*. *Parkia platycephala* was the first species to diverge in this subclade, being sister to the remaining species. *Stryphnodendron pulcherrimum* was placed as a sister to *Microlobius foetidus*. The observed topology supports previous studies of the former *Mimosoideae* subfamily [30–32] and the reclassified *Fabaceae* family [13, 15]. Therefore, through phylogenetic analyses, we strengthened the consistency of the *Caesalpinioideae* subfamily, confirming the close relatedness of both species and the accuracy of the assembled transcriptomes.

To discover common expression patterns between *P. platycephala* and *S. pulcherrimum* during species development in the *canga*, we used the gene orthology information of both species inferred by OrthoFinder and compared the single-copy orthologs expression profiles. From the 63,039 queried predicted proteins of both species, a total of 53,763 were assigned to 16,958 orthogroups. From these orthogroups, 16,010 (94.4%) were shared, 948 were classified as species-specific, and 9,284 were single-copy for both species. The single-copy shared orthogroups were used for the conserved orthologs DE analysis.

### Species-specific differential expression analysis under canga and forest substrates

To elucidate species-specific molecular mechanisms in response to the challenging *canga* environment, we identified differentially expressed genes in each species by comparing gene expression data in seedlings subjected to the *canga* substrate to those grown in the forest substrate (control). We reduced the biological variability by pooling [33] the leaf samples of three individuals grown in the same substrate for RNA extraction and sequenced two replicate pools for each condition. We also used stringent false discovery rate (FDR < 0.001, instead of the 0.05 usually found in RNA-seq studies) and log_2_FoldChange thresholds (|log_2_FC|≥ 2) to reduce the number of false-positive differentially expressed genes (DEG) detected [34]. Although this might increase the false-negative rates, it strengthens our confidence in the analyses.

**Fig. 2:**
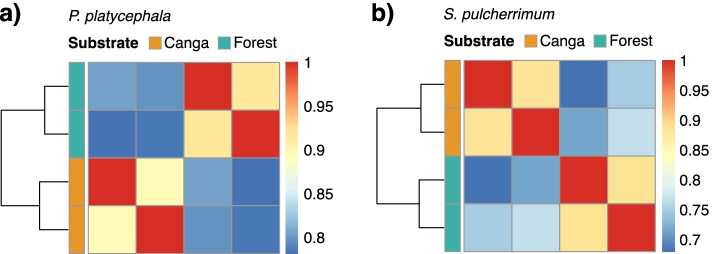
Sample clustering and correlation analysis. Symmetrical heat map showing the Spearman’s correlation coefficients of gene expression across the samples for *P. platycephala* (**a**) and *S. pulcherrimum* (**b**)*.* Correlation coefficients are shown on the left ranging from blue (low similarity) to red (high similarity)

The Spearman correlation coefficient of the expression values was calculated, showing higher correlations for the same condition than between conditions for both species (Fig. 2). The DEGs were identified using the edgeR package. We found 1,112 and 838 DEGs (FDR < 0.001) for *P. platycephala* and *S. pulcherrimum*, respectively, including 390 up-regulated and 723 down-regulated genes for *P. platycephala* and 264 up-regulated and 574 down-regulated genes for *S. pulcherrimum* (See Supplementary Tables S4 and S5, Additional File 1). Both species had more down-regulated genes in plants grown in the *canga* substrate. However, the set of differentially expressed genes varied between species, indicating species-specific responses and adaptations.

To classify the DEGs' biological function, Gene Ontology (GO) enrichment analysis was carried out using GOseq package. The GO enrichment analysis of the up-regulated genes identified 28 enriched terms (FDR < 0.05) for *P. platycephala* for the biological process (BP), cellular component (CC), and molecular function (MF) categories. For *S. pulcherrimum*, only the term “rhythmic process” was found enriched in the up-regulated genes. For the down-regulated genes, there were 85 (*P. platycephala*), and 131 (*S. pulcherrimum*) significantly enriched GO terms were detected for the BP, CC, and MF categories (See Supplementary Tables S6-S9, Additional File 1).

**Fig. 3:**
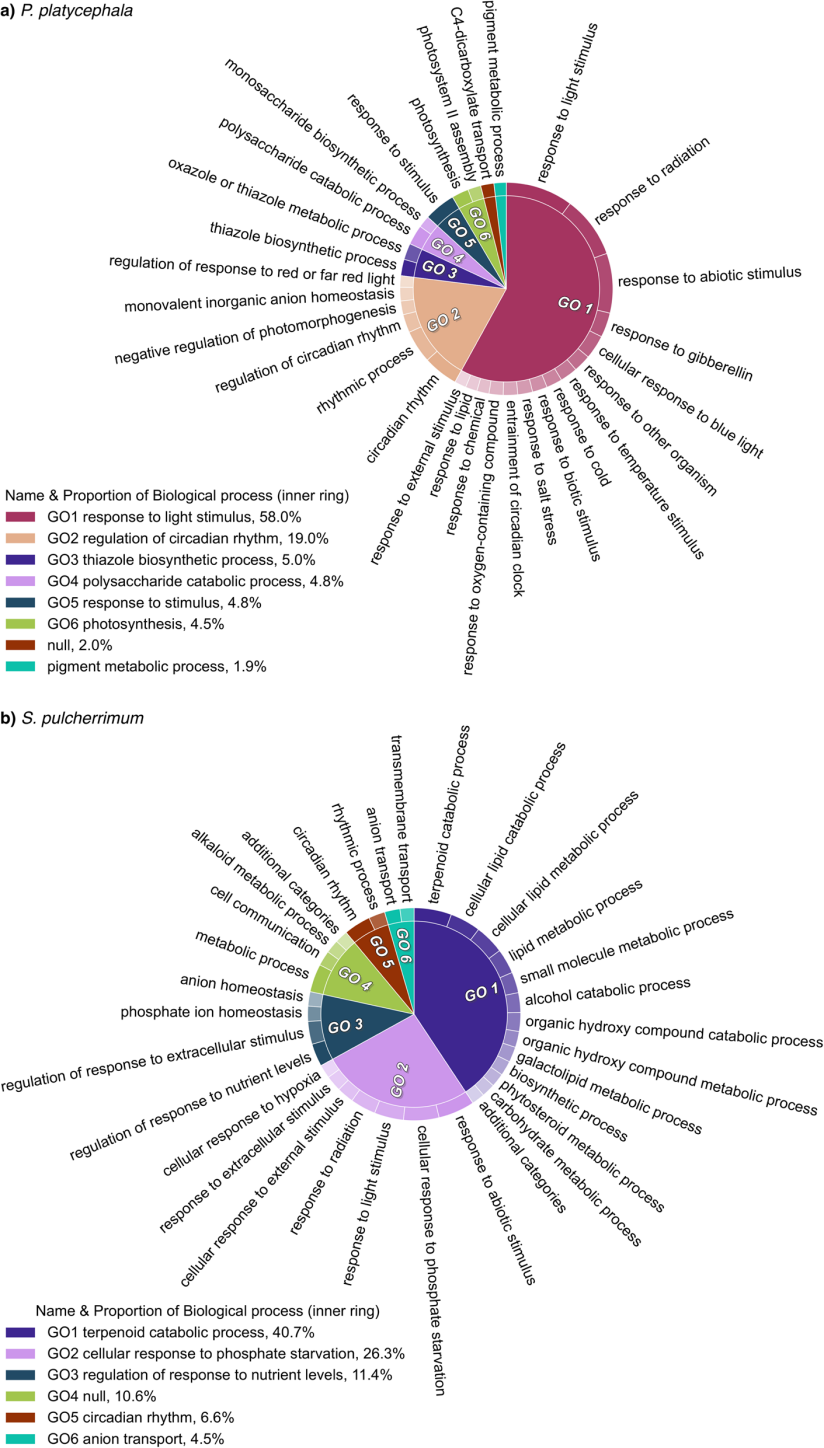
Enriched biological processes among DEGs between plants grown in *canga* and forest substrates. CirGO visualization of the top 30 GO enriched biological processes (*FDR* < 0.05) in a two-level hierarchical structure from the DEGs of (**a**) *P. platycephala* and (**b**) *S. pulcherrimum.* The legend shows the “parent” labels, represented in the pie chart's inner ring and the slice proportion. The outer ring of the pie chart represents the relative contribution of “child” labels

For *P. platycephala*, within the biological process category*,* the DEGs were mainly associated with ‘response to light stimulus’ and ‘circadian rhythm’ (Fig. 3a). The GO terms ‘rhythmic process’, ‘circadian rhythm, ‘response to (abiotic, external, light, temperature) stimuli’ were significantly enriched in up-regulated and down-regulated genes. However, the GO terms ‘oxazole or thiazole biosynthetic process’, ‘polysaccharide catabolic process’, and ‘response to biotic stimulus’ were enriched just in the up-regulated genes. The terms ‘response to gibberellin’ and ‘photosynthesis’ were enriched just in the down-regulated genes (See Supplementary Tables S6 and S7, Additional File 1). For *S. pulcherrimum,* ‘rhythmic process’ was the only enriched GO term in the up-regulated genes. The down-regulated genes were associated with the ‘circadian rhythm’, ‘terpenoid catabolic process’, ‘response to (abiotic, light, radiation) stimuli and nutrient levels’, and ‘cellular response to phosphate starvation’ (See Supplementary Tables S8 and S9, Additional File 1). Figure 3 shows the top 30 GO BP terms from the DEGs in *P. platycephala* (Fig. 3a) and *S. pulcherrimum* (Fig. 3b) grown in *canga* substrate. The complete list of enriched GO terms is in Supplementary Tables S6-S9, Additional File 1.

**Fig. 4:**
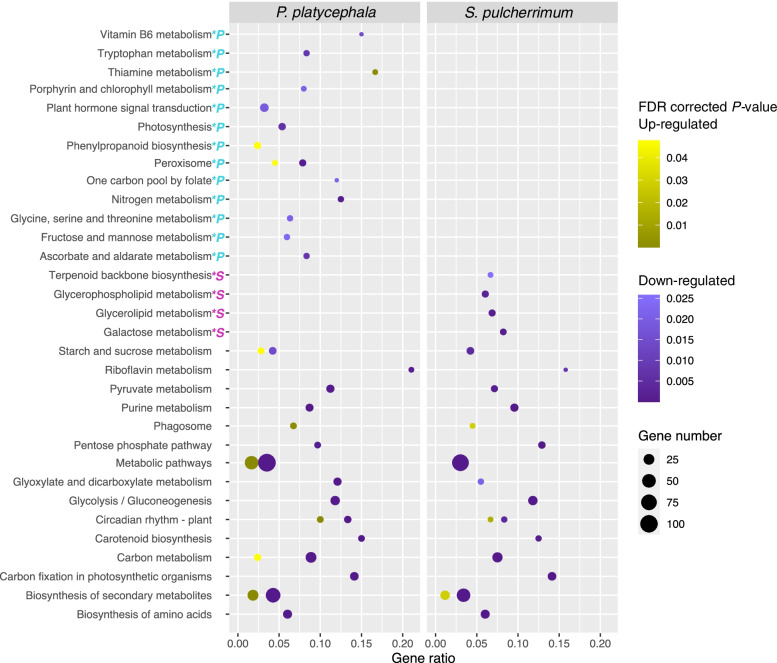
Enriched KEGG pathways among DEGs between plants grown in *canga* and forest substrates. Scatterplots of enriched KEGG pathways in up-regulated (yellow shades) and down-regulated (purple shades) gene groups for *P. platycephala* (left) and *S. pulcherrimum* (right). The significantly enriched pathways were indicated as dots (*FDR* < 0.05), and the dot sizes represent the number of the genes included in each cluster. The x-axis represents the ratio of the number of differentially expressed genes and all genes in the pathway. The deeper the color, the smaller the FDR corrected *p-*value. Colored letters next to the names of the routes indicate uniquely altered pathways in one species. P in cyan—*P. platycephala*; S in pink—*S. pulcherrimum*

To further characterize the DEGs' function, a pathway-based analysis was performed using the KEGG pathway database (https://www.genome.jp/kegg/) with KOBAS v. 3.0. We identified 28 and 19 enriched (FDR < 0.05) pathways *in P. platycephala* and *S. pulcherrimum*, respectively, 15 of which are shared between the species. Figure 4 shows all the enriched KEGG pathways from the up- and down-regulated genes for both species. Exact FDR corrected *p*-values for each enriched pathway can be found in Supplementary Tables S10-S13, Additional File 1. The enriched pathways common to both species are related to the biosynthesis of secondary metabolites and their precursors in carbohydrate metabolism and amino acid biosynthesis. Although both species showed similarly altered pathways, we also found some unique changed pathways. For *P. platycephala*, these pathways are related to the metabolism of cofactors and B-complex vitamins (porphyrin and chlorophyll; thiamine; vitamin B6; one carbon pool by folate), phenylpropanoid biosynthesis, photosynthesis, and peroxisome. For *S. pulcherrimum*, the exclusive pathways are related to lipid (glycerolipids; glycerophospholipids) and terpenoid metabolism.

### Conserved orthologs differential expression analysis under canga and forest substrates

**Fig. 5:**
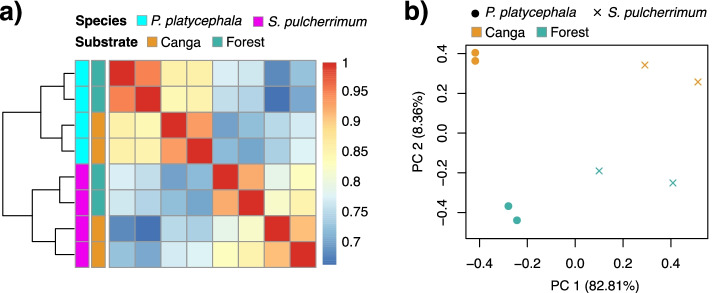
Cross-substrates comparison of ortholog expression patterns. **a** Symmetrical heat map of Spearman’s correlation coefficients between all samples across all single-copy orthologs. Correlation coefficients are shown on the left ranging from blue (low similarity) to red (high similarity) (**b**) PCA of the log-transformed normalized expression levels of the single-copy orthologs between the two species grown in the *canga* and forest substrates. Species are represented by point shape; substrates are represented by point color

To facilitate the comparison of responses between the two species and observe the existence of genes altered in the same way in the initial development of the two species in the *canga*, we retrieved 9,284 single-copy orthologs. We investigated gene expression patterns across species and conditions with a hierarchical clustering analysis based on Spearman’s correlation coefficient. Samples of the same species from different substrates clustered together, suggesting a general species-specific expression pattern of the orthologs (Fig. 5a). The principal component analysis (PCA) also revealed the species-specific expression pattern, with the first component (PC1) separating the two species (Fig. 5b). The two conditions (*canga* and forest) were separated along with the second component (PC2) (Fig. 5b).

We then looked for orthologs that were differentially expressed in the same manner in the substrates for both plant species (Similarly responding differentially expressed orthologs—SRDEOs) to identify core genes involved in adapting to the *canga* substrate. We found 298 SRDEOs; 204 were down-regulated, and 94 were up-regulated (See Supplementary Table S14, Additional File 1). The direction of gene expression of detected SRDEOs was similar to that of the separate species analyses, with more down-regulated orthologs. Enrichment analysis identified 26 enriched GO terms (FDR < 0.05—Table 2). Most terms were related to chloroplast, transcription regulation, circadian rhythm, and response to light stimulus. The enrichment analysis of the metabolic pathways showed only two enriched pathways (FDR < 0.05) in the SRDEOs: circadian rhythm and riboflavin metabolism. These results suggest that plants alter the expression of genes related to the perception of environmental variation depending on the substrate composition, which may be related to the alteration of chloroplast functions. These genes, and the other orthologs similarly altered in the two species, may represent the molecular signature of these species in the *canga* environment.

**Table 2:** Gene Ontology terms identified as enriched by Fisher's exact test among SRDEOs

GO	Category	Term	FDR	Nº of genes
GO:0009507	CC	chloroplast	1.51E-13	92
GO:0006355	BP	regulation of transcription, DNA-templated	2.76E-08	39
GO:0007623	BP	circadian rhythm	5.32E-08	12
GO:0010114	BP	response to red light	2.69E-07	9
GO:0005515	MF	protein binding	1.48E-06	60
GO:0003700	MF	DNA-binding transcription factor activity	6.36E-06	36
GO:0009649	BP	entrainment of circadian clock	1.64E-05	5
GO:0010017	BP	red or far-red light signaling pathway	1.64E-05	6
GO:0009535	CC	chloroplast thylakoid membrane	2.62E-05	14
GO:0009570	CC	chloroplast stroma	3.02E-05	19
GO:0009637	BP	response to blue light	0.00026005	6
GO:0080167	BP	response to karrikin	0.00036018	8
GO:0009416	BP	response to light stimulus	0.00053656	10
GO:0009658	BP	chloroplast organization	0.00135533	8
GO:0090351	BP	seedling development	0.00239464	4
GO:0000427	CC	plastid-encoded plastid RNA polymerase complex	0.00340051	3
GO:0032922	BP	circadian regulation of gene expression	0.00340051	3
GO:0042752	BP	regulation of circadian rhythm	0.00563682	4
GO:0042753	BP	positive regulation of circadian rhythm	0.006172	3
GO:0010218	BP	response to far red light	0.01466872	4
GO:0045892	BP	negative regulation of transcription, DNA-templated	0.01476035	7
GO:0009909	BP	regulation of flower development	0.01476035	5
GO:0009579	CC	thylakoid	0.02513588	7
GO:0009654	CC	photosystem II oxygen evolving complex	0.02601454	3
GO:0009534	CC	chloroplast thylakoid	0.03019725	7
GO:2000028	BP	regulation of photoperiodism, flowering	0.04020657	3

*BP* Biological Process, *CC* Cellular component, *MF* Molecular Function

## Discussion

*Canga* plant communities are exposed to conditions that determine severe restrictions for their establishment. Especially restrictive are high metal concentrations, radiation, temperature, and low water storage capacity [3]. Molecular adaptative mechanisms of the native flora are largely unknown. This work adds to the understanding of *P. platycephala* and *S. pulcherrimum* molecular adaptative mechanisms and the description of their gene content. This is important not only for the region where industrial mining activities occur but also because the plants have a broad distribution to other regions. We used the assembled transcriptomes as references to investigate the plants' adaptative gene expression plasticity when grown in two natural substrates. We aimed at unraveling the changes in gene expression implicated in abiotic stress compensation to the *canga* environment.

It is worth noting that, although the experiment was conducted in greenhouse conditions to diminish additional environmental variables, we collected all substrates in the field, carrying their original characteristics [19]. Organisms are exposed to multiple stressors simultaneously with potentially interactive effects. Therefore, we did not intend to decompose the soils variables and test the plants' responses to each one but to understand the overall adaptative response to the different substrates. The transcriptomic patterns shown in concurrent stresses are different from those in highly controlled conditions [25, 26, 35]. Thus, maintaining the original substrates provided a setup closer to the natural environments and better reflected the transcriptomic responses to the habitats.

*Parkia platycephala* and *S. pulcherrimum* exhibited a similar direction of the altered transcriptional state, with more down-regulated than up-regulated genes, when grown in the *canga* substrate. The functional analysis indicated alterations in the metabolic pathways related to both species' primary and secondary metabolite synthesis. Approximately 52% and 75% of the altered pathways in *P. platycephala* and *S. pulcherrimum*, respectively, were shared between species, most related to primary metabolism, but with possible impact in secondary metabolites [27, 36]. The pathways related to secondary metabolites differ in the two species, indicating that the species have different survival strategies. *Parkia platycephala* seems to direct the changes in gene expression mainly through the shikimate (shikimic acid) pathway, produced from the glycolytic and pentose phosphate pathways (enriched in the analysis). This pathway produces phenylalanine, tyrosine, tryptophan (the last two enriched in the analysis), precursors of several secondary metabolites, including phenylpropanoids [36]. *Stryphnodendron pulcherrimum* seems to direct the changes to the mevalonate pathway, which culminates in the production of lipids and terpenoids [36], over-represented in enrichment analyzes.

Phenylpropanoids are a group of secondary plant metabolites derived from phenylalanine that have various functions both as structural and signaling molecules [37]. Thirteen over-expressed genes in *P. platycephala* in the *canga* soil were related to the phenylpropanoid biosynthesis, including the monolignol biosynthesis, the starting compounds for lignin biosynthesis, a key structural organic polymer for plant growth and development [38]. Lignin confers cell wall rigidity, providing structural support and acting as a barrier against pathogens. This plant polysaccharide can also be involved in mineral nutrition and the plant's response to various environmental stresses, such as the tolerance of drought, heat, and heavy metals [38–41], all observed in *canga* environment. Therefore, it could be acting as one of the physiological mechanisms involved in *P. platycephala* metal tolerance. Silva and collaborators [19] previously observed high Zn (zinc) and Mn (manganese) availability in the substrate, together with elevated concentrations of Mn and Fe (iron) in the leaf tissues. Such lignin involvement in metal tolerance has also been reported for the Mn-hyperaccumulator *Phytolacca americana* [40]. The sequestration of Mn into the leaf cell wall was also found to contribute to Mn tolerance in the sugarcane [42].

In addition, the up-regulated genes related to the phenylpropanoid biosynthesis pathway included peroxidase and cinnamyl-alcohol dehydrogenase, involved in the regulation of reactive oxygen species (ROS) levels [43]. ROS can change the integrity of cell structure and lead to the denaturation of functional and structural proteins and lipid deterioration [44]. Other *P. platycephala* altered pathways related to ROS regulation were peroxisome, ascorbate, alderate, and glutathione metabolisms. We observed DEGs coding for ROS scavenging enzymes, such as glutathione S-transferases, catalase (up-regulated), ascorbate peroxidase, and iron superoxide dismutase (down-regulated). The enzyme sarcosine oxidase, which produces glycine, formaldehyde, and hydrogen peroxide (H_2_O_2_) in peroxisomes, was also found DE in *canga* plants.

*Parkia platycephala* exhibited down-regulation of genes related to photosynthesis and carbon fixation when cultivated in the *canga* soil. The downregulation of genes coding for photosynthetic proteins has been frequently observed in abiotic stresses, such as drought, salt, temperature, and heavy metals [45–47]. High metal concentration in the photosynthetic tissue may reduce the synthesis of photosynthetic pigments and damage the photosynthetic machinery [48]. The mechanisms adopted by the plants in this study are primarily related to the negative consequences on chlorophyll biosynthesis, the formation of the photosystems, and electron transport mechanisms. Genes related to the carbon fixation pathway were also down-regulated in *S. pulcherrimum* in *canga.* Despite the observed modulation in gene expression, a significant reduction in biomass accumulation or growth performance was not observed between the substrates [19].

Genes involved in the terpenoid backbone biosynthesis were found down-regulated in *S. pulcherrimum*. Terpenoids are the largest class of secondary metabolites in plants and play essential roles in relieving abiotic and biotic stresses [49]. The altered genes in this pathway code for enzymes that catalyzes reactions releasing pyrophosphate (Geranylgeranyl pyrophosphate synthase, Dehydrodolichyl diphosphate synthase complex, Solanesyl-diphosphate synthase 2, Geranylgeranyl diphosphate reductase). Phosphorus (P) concentration was higher in the *canga* substrate [19]. Moreover, forest grown *S. pulcherrimum* showed lower leaf P content [19]. The higher expression of genes related to terpenoid biosynthesis observed in plants grown in forest substrate may be related to the low P availability. Phosphorus influences terpenoid production since its synthesis depends on ATP and NADPH, and terpenoid precursors contain high-energy phosphate bonds [50, 51]. Therefore, we suggest that terpenoids may act as phosphate providers under P-limiting conditions.

Enrichment analyzes indicated a response of *S. pulcherrimum* to P deprivation in the forest substrate with 15 GDEs associated with the GO term ‘response to phosphate starvation’. The expression of genes associated with the synthesis of monogalactosyldiacylglycerol (MGDG), digalactosyldiacylglycerol (DGDG), and sulfoquinovosyldiacylglycerol (SQDG) was higher in plants grown in forest substrate. These compounds are galactolipids (MGDG and GDGD) and sulfolipids (SQDG) that constitute most of the chloroplast membrane lipids (15% are phospholipids), making the organelle minimally dependent on phosphate [52]. Pathways related to lipid and galactose metabolism were also enriched in the analysis. During exposure to phosphorus deprivation, plants reallocate phosphate through the exchange of chloroplast membrane lipids [53, 54]. The biosynthesis of galactolipids and sulfolipids is increased, replacing phospholipids and releasing phosphate to maintain nucleic acid levels and metabolic activity [52–54]. Thus, despite phosphate deficiency, *S. pulcherrimum* appears to have thrived on the forest substrate through phosphorus reallocation from chloroplast membrane lipids and terpenoid precursors molecules.

Harsh environmental conditions limit the range of ecological strategies and lead to trait convergence. This convergence may or may not be the result of the expression of the same set of genes [55]. Thus, we evaluated if the studied species show conserved gene expression responses underlying adaptations to the substrates. The comparative ortholog transcriptomic analysis revealed that the expression patterns differed more between species than between substrates, indicating that the overall gene expression pattern is organism-specific. Still, almost 300 pairs of orthologous genes in *P. platycephala* and *S. pulcherrimum* were observed with similar expression changes during development in *canga* and forest substrates. These transcripts code for proteins involved in the plant circadian rhythm. The circadian rhythm pathway and GO terms were also enriched in the species-specific analysis in the two conditions. The circadian rhythm is known to be synchronized by changes in light and temperature stimuli [56, 57]. It allows plants to anticipate daily and seasonal changes in the environment essential to regulate their growth and survival [58]. Several studies have demonstrated that the circadian clock contributes to the plants’ ability to tolerate and thrive despite a wide spectrum of stress signals, including iron deficiency, alkaline stress, and drought or salinity stress [59, 60]. In this study, the growth in *canga* soil down-regulated the expression of circadian rhythm genes: *NIGHT LIGHT-INDUCIBLE AND CLOCK-REGULATED* (*LNKs 1, 2,* and *3*), *UNE10* (also known as *PIF8*), and *LATE ELONGATED HYPOCOTYL (LHY)*, expressed in the morning, while up-regulating evening-expressed genes (*PCL1, EARLY FLOWERING 4—ELF4, PSEUDO-RESPONSE REGULATOR—PRR5*), and *CORs* (27 and 28) orthologs in both species. The pattern of clock gene expression observed here seems to be related to the down-regulation of the chloroplast functions. Circadian regulation is integrated with photosynthesis, carbon fixation metabolism, and its metabolic products [61, 62]. *CCA1*, an *Arabidopsis LHY* homologous, was found to increase activity in response to sugar. On the other hand, *PRR7* was found to be repressed [61]. Indeed, pathways related to carbon metabolisms such as carbon fixation in photosynthetic organisms, glycolysis, and pentose phosphate pathways, were enriched in the down-regulated genes in both species in the species-specific analyses. Therefore, the substrate composition affected the expression of the circadian rhythm genes in the leaves, regulating the carbon fixation metabolism. This might provide adaptations to optimize plant performance in environments with different nutritional conditions.

Another possibility is that the studied plants altered the circadian clock phase by advancing the expression of evening-expressing genes, as the biological samples were collected in the morning. A similar advance of the circadian clock phase was also observed in barley under osmotic stress [63]. The circadian clock has a role in micronutrient homeostasis regulation in plants, including acquisition and transport to the shoots [64–66]. Chen and collaborators [66] showed that Fe deficiency lengthened the circadian rhythm period in *Arabidopsis*. Both *P. platycephala* and *S. pulcherrimum* in the *canga* substrate exhibited high Fe concentration in the shoots [19] that may be related to a shortened circadian period which may explain the early expression of the evening genes. Observing the circadian rhythm genes expression at various time points during the day may elucidate if the *canga* substrate shortens the circadian period. Overall, many genes involved in the abiotic stress response are under the control of the circadian rhythm, even for environmental conditions that are constant in a diurnal manner, such as drought and salinity [58, 60]. Our results suggest that plants adapted to both *canga* and forest environments can modulate the circadian rhythm in a substrate-dependent manner that might help them thrive in this range of conditions. The circadian clock is conserved among living species since it controls general metabolic processes and ensures plants' acclimation to their environment. One interesting possibility is investigating if *canga* endemic plants present diminished circadian rhythm plasticity, limiting their capacity to strive in different environments. The modification of the circadian clock genes may enhance crop growth and yields [67, 68]. Here we show that the modulation of circadian clock genes may improve local environment adaptation by nutritional status perception.

## Conclusion

The *canga* environment presents numerous plant stressors and, despite the harsh conditions, many plant species are well adapted and capable of thriving in this environment. In this study, we identified DEGs in two *Fabaceae* species, capable of inhabiting the forest and the *canga* environments, that were grown under both substrate conditions. We observed that the substrate modulates gene expression in *P. platycephala* and *S. pulcherrimum*. Both species exhibited changes in major metabolic pathways, such as biosynthesis of secondary metabolites and carbon metabolism, and adopted species-specific strategies for adaptation in the *canga* environment. Genes involved in plants’ response to environmental stimuli, such as phenylpropanoid biosynthesis and photosynthesis, were altered in *P. platycephala*. *Stryphnodendron pulcherrimum* specific DEGs were associated with the phosphate deprivation response. Our results also show evidence that the studied species exhibit shared adaptative transcriptional responses to the *canga* environment. The modulation of circadian rhythm genes was a common mechanism related to the *canga* environment that forced a similar expression for each species.

## Methods

### Biological material and experimental design

The leaf samples used in this research were obtained from the plant physiology study by Silva and collaborators (2018) [19]. *P. platycephala* and *S. pulcherrimum* were grown in four substrates collected from the Carajás Mineral Province, Pará, Brazil. The region is rich in iron ore deposits (with active mining activities). An area of *canga*, an adjacent forest, and two mine wastes soil substrates (Red waste and Yellow waste) were chosen for substrate collection. A detailed description of the site and soil types is available in Silva et al., 2018 [19]. In summary, the seeds from *P. platycephala* and *S. pulcherrimum* were obtained from Vale’s tree nursery in Carajás. Five days after germination in Petri dishes, ten seedlings of each species were planted in 35 cm × 24 cm × 18 cm (L × W × H) trays containing 12 L of one of the four substrates. For the RNA-seq experiment, we used two trays for each substrate. After 45 days, we harvested the fully expanded leaves from *P. platycephala* and *S. pulcherrimum* under each condition. Samples were fast-frozen in liquid nitrogen and stored at -80 ºC for posterior RNA extraction. Leaves from three seedlings in the same tray were pooled together and considered one composite replicate for each species. All the samples were harvested during morning time (09:30–10:30 local time) to minimize the diurnal differences. The experiment was conducted in a greenhouse. The temperature varied from 25 to 30ºC, and the midday photosynthetic photon flux density (PPFD) was 1,500 μmol m-2 s-1. Water availability was maintained at 70% of the soil water retention capacity by daily irrigation after trays weighting to determine water loss.

In the present study, we aimed to assemble high-quality and complete transcriptomes to use as a reference for *P. platycephala* and *S. pulcherrimum*. For that, we performed the RNA extraction, sequencing, and transcriptome assemblies with samples of both species grown in all four substrates. Furthermore, we sought to reveal the gene expression plasticity of the plants grown in substrates where they naturally occur. Therefore, we performed the remaining analysis (Species-specific differential expression [DE] analysis and DE analysis of the orthologs between the species) only with the plants’ samples grown on the *canga* and forest substrates. We used the plant samples from the forest substrate as a control.

### RNA isolation, library preparation, and sequencing

Leaf samples were ground in liquid nitrogen. Total RNAs were extracted using the RNeasy mini kit (QIAGEN) following the manufacturer’s protocols. RNA integrity and concentration were determined using a 2100 Bioanalyzer (Agilent Technologies, Waldbronn, Germany) and a Qubit® RNA High Sensitivity Assay Kit (Thermo Fisher Scientific, Waltham, MA, USA), respectively. Only RNAs with RIN (RNA Integrity Number) greater than 8 were used for the next steps. For each sample, 100 ng of total RNA from each sample was used in the Ribo-Zero rRNA removal kit (Illumina, San Diego, CA). Subsequently, the rRNA-depleted RNA was processed using the TruSeq Stranded RNA library preparation kit (Illumina, San Diego, CA). The libraries were evaluated using Qubit® DNA Broad-range Assay Kit and 2100 Bioanalyzer. Individual libraries were uniquely barcoded, multiplexed, and paired-end sequenced (2 × 150 bp) on the Illumina NextSeq 500 sequencer (NextSeq 500 Control Software v.4.0.2) at the Instituto Tecnológico Vale, Belém, Pará, Brazil with the High Output kit v.2.5 (300 cycles). All raw reads generated from this study were deposited in the Short Read Archive (SRA) of NCBI under accession number PRJNA645405.

### Transcriptome assembly

Raw reads were evaluated for quality using the FastQC v. 0.11.5 program [69] and processed to filter Illumina adapters, and low-quality reads using Trimmomatic v. 0.38 [70]. Orphaned reads were assigned as single-end reads. The reads were also aligned to the Silva and RFAM databases to subtract rRNA reads that passed the rRNA depletion protocol. Since each assembler and parameter set produces distinct sets of high-quality transcripts, several assemblies were generated to select the best set of recovered transcripts, as recommended by Gilbert (2013) [71]. Therefore, to produce the most accurate plants gene sets possible and to maximize the diversity and completeness of de novo assembled transcripts, the remaining clean reads were used to assemble transcripts with four different assemblers: Trinity v.2.8.3 [72], rnaSPAdes v. 3.12.0 [73], Velvet v. 1.2.10/Oases v. 0.2.09 [74], and SOAPdenovoTrans v. 1.03 [75]. Kmergenie v. 1.7039 [76] was used to calculate the probable best k-mer size for assembling from the union of all libraries left reads, all right reads, and the union of all reads. Trinity was used with k-mer 25, rnaSPAdes was used with default parameters since it calculates internally the best k-mer size. Velvet/Oases and SOAPdenovo-Trans were used with multiple k-mers, ranging from 21 to 81 with a step size of 10 and the resulting k-mers from Kmergenie (*P. platycephala* – 31, 51 and 57; *S. pulcherrimum* – 27, 31 and 39). The cleaned paired-end reads were used for all assemblies, except for those generated with SOAPdenovo-Trans, which also included the cleaned single-end reads. All programs parameters used are available in Supplementary Table S15, Additional File 1.

The resulting assemblies from each assembler were combined into one merged assembly for each species and processed to mitigate redundancy with the EvidentialGene tr2aacds v.4 pipeline [71] (http://arthropods.eugenes.org/EvidentialGene/about/EvidentialGene_trassembly_pipe.html). The tr2aacds pipeline selects the best set of assembled transcripts from the input assembly based on coding potential. This pipeline uses several programs to 1) remove perfect redundancy (fastanrdb/exonerate-2.2.0), 2) remove perfect fragment (CD-HIT-EST), and 3) find high-identity exon-sized alignments (blastn) and output transcripts into three classes: okay (the best transcripts with the unique CDS), okalt (alternative transcripts, possible isoforms), and drop (the transcripts that did not pass the internal filter). Subsequently, this new program version does the second stage of analysis over the initial classes and reclassifies them into drop and okay. The okay set was selected for the subsequent analysis, including the main transcripts with alternates and those with no alternates.

As a measure of assembly accuracy, the percentage of correctly assembled bases was obtained by mapping Illumina reads back to the initial transcripts using Bowtie2 v. 2.23 [77]. The quality and completeness of the transcriptome can have a substantial impact on annotation and other downstream analyses. Errors in the transcriptome assembly could affect ortholog prediction, phylogenetic signal, and gene expression quantification [78, 79]. Therefore, the completeness of the assembled transcriptomes was assessed using BUSCO v.3.0.2 [80] to obtain the percentage of single-copy orthologs represented in the embryophyta_odb9 and eudicotyledons_odb10 databases.

### Annotation

The translated coding sequences produced from EvidentialGene were functionally annotated using the Trinotate v.3.2.1 [81]. Homology searches were performed using BLASTp [82] against the UniProtKB/Swiss-Prot database, with an e-value of 1e10^−6^, and hmmer v.3.1b2 (http://hmmer.org) against the common protein domains of Pfam database [83]. Transmembrane regions were predicted using the tmhmm v.2 [84] server, and ribosomal RNA genes were detected with RNAMMER v.1.2 [85]. Annotation outputs were loaded into a Trinotate SQLite Database.

### Assembly filtering

According to Gilbert (2019) [86], short putative proteins are spurious loci that can be discarded if no further classification evidence is established. Therefore, the Evigene draft transcripts sets for *P. platycephala* and *S. pulcherrimum* were filtered to discard possibly spurious loci. For this purpose, putative proteins shorter than 120aa long from the main set of transcripts were blasted (e-value 1 × 10^–6^) against the UniProtKB/Swiss-Prot *Viridiplantae* database. The contigs codifying the short putative proteins with homology to the *Viridiplantae* database, the contigs codifying putative proteins longer than 120aa, and their alternative forms were used for differential expression (DE) analysis in each species.

### Gene orthology prediction

The OrthoFinder v. 2.3.12 [87, 88] software was used to identify the two species' orthologous groups of protein sequences. Identification of orthogroups was conducted utilizing the Evigene predicted amino acid sequences after the above filtering step and classified as main or noclass (i. e., not the alternate forms) since the authors recommend using a single representative transcript-variant for each gene in the analysis.

It is known that the quality of the transcriptome assembly affects phylogenetic inferences and that high-quality assemblies contribute to the greater consistency of established phylogenies [79]. Thus, to assess the molecular phylogeny of the *Caesalpinioideae* subfamily, transcriptome assemblies of *Caesalpinioideae* species were downloaded from various sources (See Supplementary Table S3, Additional File 1). The data included all the assemblies used by Koenen and collaborators (2020) [13] and more recent deposits from the NCBI Transcriptome Shotgun Assembly. The amino acid sequences were extracted from the transcriptome assemblies with EvidentialGene, and only the main set of transcripts were used as input for OrthoFinder. The orthogroups of the 18 species used (including *P. platycephala* and *S. pulcherrimum*) were identified. The species tree was inferred from multiple sequence alignments, generated with 331 single-copy orthogroups, with one sequence present in at least 14 (77.8%) of the 18 species analyzed. We constructed the phylogenetic tree using the approximate maximum likelihood method in the FastTree 2 package [89] with local bootstrap values of 1,000 replicates. The tree was rooted with *Acrocarpus fraxinifolius* as an outgroup by the STRIDE algorithm [90]. MAFFT [91] was used to generate the multiple sequence alignments.

### Differential expression analysis

For each species, the number of transcripts expressed in plant growth in both *canga* and forest soil types was quantified by mapping each condition's cleaned reads to the respective filtered transcriptome assembly using RSEM v. 1.3.0 [92] and Bowtie2 v. 2.23. We then calculated Spearman’s correlation coefficients between all replicates using gene expression data. The DE analyses were performed using the estimated counts generated from RSEM with the edgeR package [93]. Computed *p*-values were corrected for multiple comparisons with the Benjamini–Hochberg procedure. A false discovery rate (FDR) cutoff value of 0.001 and a log_2_ Fold Change (log_2_FC) ≥ 2 were considered to classify transcripts as differentially expressed in the species-specific analysis.

To the cross-species comparison, DE analysis was performed just with the orthologous genes shared between the two species obtained with OrthoFinder, as proposed by Moreno-Santillán and collaborators (2019) [94]. In brief, each biological replicate of each condition was aligned to its transcriptome with RSEM. The quantification files were edited to replace the transcript IDs generated by EvidentialGene, for its respective Single Gene Orthogroup name. The quantification files were concatenated to a single count matrix. To find orthogroups that behave similarly in plants under the different substrates, we performed DE analysis with the count matrix and the edgeR method using two-factor Generalized Linear Models (GLMs). The GLMs were implemented in edgeR using an additive model to correct the difference in gene expression between species and obtain differentially expressed genes between conditions. *P*-values were corrected for multiple testing with the Benjamini–Hochberg procedure. glmTreat was used to classify orthologs as differentially expressed above Fold Change 2.

Gene Ontology (GO) enrichment analysis and Kyoto Encyclopedia of Gene and Genome (KEGG) pathway analysis were implemented by the GOseq R package [95] and KOBAS software [96], respectively, with *Medicago truncatula* as the background species for the KEGG analysis. GOs and pathways with FDR corrected *p*-value < 0.05 were considered significantly enriched. Significant GO annotations were processed with REVIGO [97] to summarize the main annotations and remove redundancy. The REVIGO output was then fed as input to CirGO v. 1.0 [98] for pie chart visualization. The GOChord function of the R package GOplot [99] was also used to visualize the enriched GO terms in the DE orthologs. The R package ggplot2 [100] was used for the enriched KEGG pathways visualization.

## Supplementary Information

**Additional file 1: Table 1. **Summary of sequencing and quality filtering. **Table 2.** Summary of assemblies. **Table 3.** The source of the species used in the phylogenetic analysis. **Table 4.** List of differentially expressed genes of P. platycephala in canga vs. forest. **Table 5.** List of differentially expressed genes of S. pulcherrimum in canga vs. forest. **Table 6.** The result of GO enrichment analysis for up-regulated DEGs in P. platycephala. **Table 7.** The result of GO enrichment analysis for down-regulated DEGs in P. platycephala. **Table 8.** The result of GO enrichment analysis for up-regulated DEGs in S. pulcherrimum. **Table 9.** The result of GO enrichment analysis for down-regulated DEGs in S. pulcherrimum. **Table 10.** The result of KEGG pathway enrichment test for up-regulated DEGs in P. platycephala. **Table 11.** The result of KEGG pathway enrichment test for down-regulated DEGs in P. platycephala. **Table 12.** The result of KEGG pathway enrichment test for up-regulated DEGs in S. pulcherrimum. **Table 13.** The result of KEGG pathway enrichment test for down-regulated DEGs in S. pulcherrimum. **Table 14.** List of differentially expressed orthogroups of plants in canga vs. forest. **Table S15.** Programs parameters.

## Data Availability

The raw datasets generated and analyzed during the current study are available in the NCBI Sequence Read Archive (SRA) repository under the Bioproject number PRJNA645405. The datasets supporting the conclusions of this article are included in this article and its supplementary information files.
